# GATA2 as a potential metastasis-driving gene in prostate cancer

**DOI:** 10.18632/oncotarget.1296

**Published:** 2014-01-12

**Authors:** Yan Ting Chiang, Kendric Wang, Ladan Fazli, Robert Z. Qi, Martin E. Gleave, Colin C. Collins, Peter W. Gout, Yuzhuo Wang

**Affiliations:** ^1^ Department of Experimental Therapeutics, BC Cancer Research Centre, Vancouver BC, Canada; ^2^ The Vancouver Prostate Centre, Vancouver General Hospital and Department of Urologic Sciences, the University of British Columbia, Vancouver BC, Canada; ^3^ Division of Life Science, the Hong Kong University of Science and Technology, Hong Kong

**Keywords:** GATA2 gene, prostate cancer, metastasis, focal adhesion, master regulatory gene

## Abstract

Effective treatment for metastatic prostate cancer is critically needed. The present study was aimed at identifying metastasis-driving genes as potential targets for therapy (oncotargets). A differential gene expression profile of metastatic LTL-313H and non-metastatic LTL-313B prostate cancer tissue xenografts, derived from one patient's specimen, was subjected to integrative analysis using the Ingenuity Upstream Regulator Analysis tool. Six candidate master regulatory genes were identified, including GATA2, a gene encoding a pioneer factor, a special transcription factor facilitating the recruitment of additional transcription factors. Elevated GATA2 expression in metastatic prostate cancer tissues correlated with poor patient prognosis. Furthermore, GATA2 gene silencing in human prostate cancer LNCaP cells led to a marked reduction in cell migration, tissue invasion, focal adhesion disassembly and to a dramatic change in cell transcriptomes, indicating that GATA2 plays a critical role in prostate cancer metastasis. As such, GATA2 could represent a prostate cancer metastasis-driving gene and a potential target for therapy of metastatic prostate cancer.

## INTRODUCTION

Prostate cancer is the most commonly diagnosed non-cutaneous cancer and the second leading cause of cancer death for North American men [1]. When the malignancy is localized to the prostate, surgery and radiation therapy can be curative. However, many treated patients will experience local recurrence or metastasis [2–4]. Advanced, metastatic prostate cancer is highly resistant to conventional therapy and is currently incurable. Discovery of new therapeutic targets for more effective treatment of metastatic prostate cancer is urgently needed for improved disease management and patient survival [5–8].

Metastasis is a multi-step process of complex, interrelated events, including cell detachment from the primary tumour, tissue invasion, survival in blood or lymph vessels, extravasation and adhesion and proliferation at a distant site [9–11]. Metastasis is generally thought to result from changes in the expression of specific, master regulatory genes that lead to cascades of downstream genes mediating the metastatic process. Such metastasis-driving genes could serve as therapeutic targets for management of metastatic prostate cancer [12–14]. In trying to identify such genes, approaches have in general been based on identification of the highest differentially expressed genes in metastatic versus non-metastatic cancer cells [15–17]. However, gene regulatory networks often act as amplification cascades. In such a case, the highest differentially expressed genes would represent downstream genes and not upstream, metastasis-driving genes, since the latter would show smaller changes in gene expression. Recently, it has become possible to predict upstream driver genes through integrative, software-based analysis of differential gene expression profiles coupled to information of upstream regulatory genes obtained from molecular studies [18–21].

The *GATA2* gene is one of the six members of the GATA transcription factor gene family that regulates cellular differentiation [22]. It is known as the master regulator in the development of the hematopoietic system [23, 24]. Recently, GATA2 protein has been reported as the predominant GATA factor expressed in normal human and mouse prostate [25]. However, a role for GATA2 in the development of metastatic prostate cancer has not been reported.

Metastasis-driving genes may be identified by integrative analysis of gene expressions of metastatic and non-metastatic cancer cells. In the present study, we analyzed a differential gene expression profile of paired metastatic and non-metastatic prostate cancer tissue xenograft lines derived from one patient's primary tumor [26, 27]. Six candidate genes were identified, including the *GATA2* gene. *In vitro* evidence that *GATA2* plays a role in prostate cancer metastasis, and the finding that its elevated expression in clinical metastatic prostate cancer tissues correlates with poor patient prognosis, suggest that the *GATA2* gene is a potential prostate cancer metastasis-driving gene.

## RESULTS

### GATA2 as a potential upstream master regulatory gene

**Figure 1:**
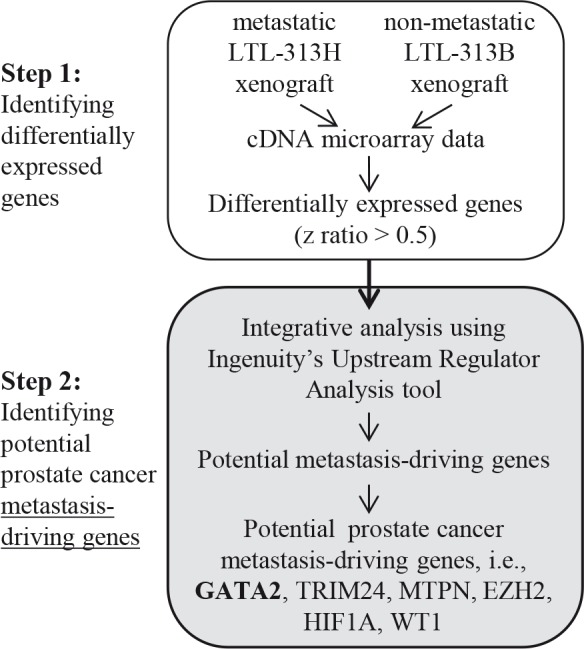
Two-step strategy used in identifying potential metastasis-driving genes in prostate cancer Differentially expressed genes with z ratio of > 0.5 were identified by comparing microarray gene expressions from paired metastatic LTL-313H and non-metastatic LTL-313B prostate cancer tissue xenografts. The differential gene expression profile was then analyzed using IPA's Upstream Regulator Analysis tool, in combination with reported, relevant molecular data, to predict potential metastasis-driving genes (see Supplementary Table 1). A number of potential prostate cancer metastasis-driving genes were identified including *GATA2*.

Using previously obtained microarray gene expression data (GSE41193) from paired metastatic LTL-313H and non-metastatic LTL-313B prostate cancer xenografts [27], approximately 700 differentially expressed genes (with a z ratio > 0.5) were identified. Analysis of these genes using the Ingenuity Upstream Regulator Analysis tool pinpointed 18 potential upstream master regulatory genes, as shown in Supplementary Table 1. This number of genes was subsequently narrowed down by excluding genes that are not expressed in prostate tissue or genes that showed down-regulated expression in metastatic prostate cancer patients' specimens [28, 29]. As presented in Figure 1, the following potential prostate cancer metastasis-driving genes were identified: *GATA2, TRIM24, MTPN, HIF1A, WT1*, and *EZH2*. The *GATA2* transcription factor gene was of particular interest since, as a pioneer factor in prostate cancer, it has a potential role in cellular reprogramming and hence in the development of metastasis [30–32].

### Elevated expression of GATA2 correlates with poor prostate cancer patient prognosis

**Figure 2:**
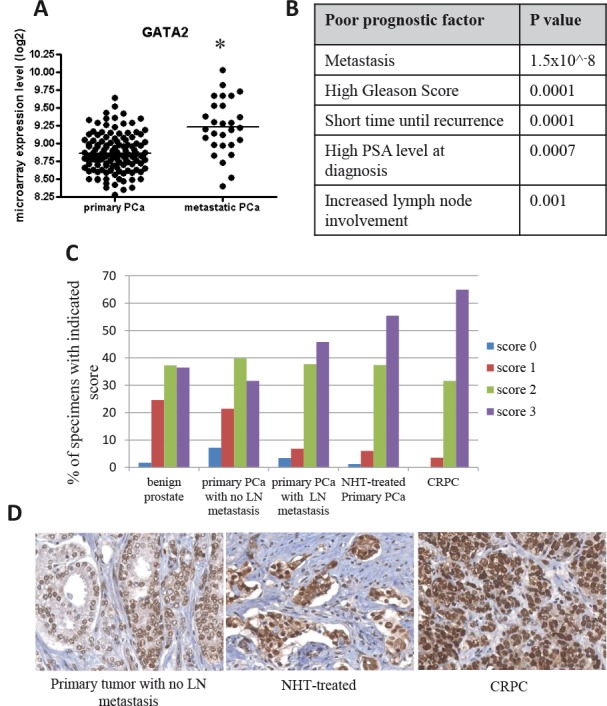
Elevated expression of GATA2 associated with poor prostate cancer (PCa) patient prognosis A, *GATA2* is highly expressed in 19 metastatic PCa tissues compared to 131 primary PCa samples (microarray gene expression data from the MSKCC Prostate Oncogenome Project) (*, p < 0.001). B, elevated expression of *GATA2* is associated with poor patient prognosis with the indicated p value. The p values were calculated using the Student's t-test. C, increased GATA2 immunostaining intensity was observed in advanced prostate cancers. Specimens with scores 0–3 are presented as percentages of 359 samples. D, from left to right, representative images from a primary tumor with no lymph node (LN) metastasis, a neoadjuvant hormonal therapy (NHT)-treated primary tumor (1–12 months), and a castration-resistant prostate cancer (CRPC).

Examination of a large scale, integrated cancer genomic dataset of the MSKCC Prostate Oncogenome Project [28] indicated that *GATA2* gene expression was significantly elevated in metastatic prostate cancer samples (Fig. 2A). Elevated *GATA2* gene expression also correlated with shorter times of disease recurrence, increased lymph node involvement, increased Gleason score and elevated PSA levels at diagnosis (p<0.05; Fig. 2B). As shown in Figure 2C, a similar correlation was found between elevated GATA2 protein expression and malignant progression of prostate cancer, as shown for clinical prostate cancer samples with (i) increased lymph node involvement, (ii) following neo-adjuvant treatment and (iii) development of castration resistance.

### GATA2 gene silencing reduces *in vitro* proliferation, migration and matrigel invasion of prostate cancer cells

**Figure 3:**
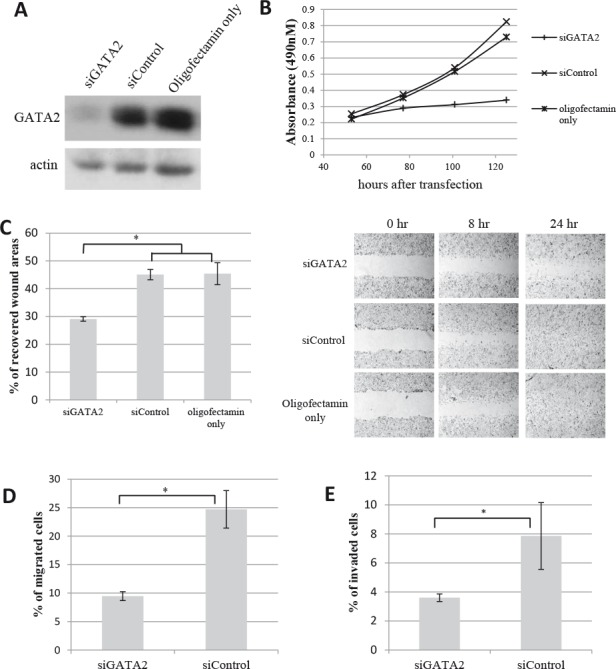
Knock-down of GATA2 gene expression decreases proliferation, migration and matrigel invasion of prostate cancer cells Treatment of LNCaP cells with siGATA2 leads to A, a marked reduction in GATA2 protein levels; and B, a marked decrease in cell proliferation. C, a monolayer of LNCaP cells was scratched to examine the rate of cell migration into the wounded area. The bar graph represents the percentage of cell-recovered wound areas after 8 hours of incubation (*, p<0.01). Representative images of the wound captured at different time points are shown (at right). D & E, cell migration and matrigel invasion assays show a marked decrease in cell motility and tissue invasiveness of siGATA2-treated LNCaP cells. Bar graphs show the percentage of migrated/invaded cells after a 20-hr incubation. Results (A-E) shown are representative of three individual experiments with error bars representing standard deviation based on triplicates. Statistical significance was established using the Student's t-test.

siRNA-induced silencing of *GATA2* gene expression in LNCaP cells led to a very marked reduction in GATA2 protein levels (Fig. 3A), and greatly inhibited cell proliferation (Fig. 3B). Similar results were found with C4-2 cells (Supplementary Fig. 1A). Furthermore, *GATA2* silencing significantly reduced LNCaP cell motility as revealed by an 8-hr wound healing assay (Fig. 3C). The reduced cell motility does not appear to be a consequence of reduced cell proliferation since the doubling time of LNCaP cell cultures is about 48 hours. As well, Boyden chamber assays showed that *GATA2* silencing markedly reduced both migration (Fig. 3D) and tissue invasion (Fig. 3E) of the cells.

### A role for the GATA2 gene in focal adhesion disassembly

**Figure 4:**
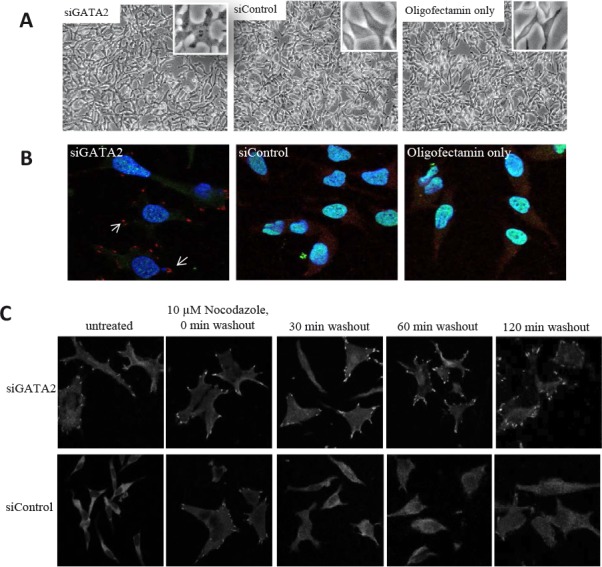
A role for the GATA2 gene in focal adhesion disassembly A, following treatment with siGATA2, the smooth edge spindle-like LNCaP cells became flat and developed visible focal contacts. B, siGATA2-treated cells showed a clear focal adhesion immunostaining pattern in cells. Cells were immunostained with anti-vinculin-TRITC (red), anti-GATA2-FITC (green), and DAPI (blue). C, treatment with siGATA2 led to a failure in the disassembly of focal adhesions. Serum-starved siControl and siGATA2-treated LNCaP cells were incubated with 10 μM nocodazole for 4 hours. Cells at the indicated times after nocodazole washout were fixed and immunostained with anti-vinculin. Images were taken at 63x magnification.

Silencing of the *GATA2* gene in LNCaP cells induced a number of morphological changes. The normally smooth edged, spindle-like LNCaP cells became flat and developed focal contacts at the cell edges (Fig. 4A). Similar morphological changes were found for C4-2 cells (Supplementary Fig. 1B). In LNCaP cells that were immunofluorescence-stained for the focal adhesion protein, vinculin [33], focal adhesion complexes were observed in > 80% of *GATA2*-silenced cells, whereas < 5% of the control cells showed such complexes (Fig. 4B). We also checked the effect of *GATA2* silencing on focal adhesion disassembly since enhancement of this process is critically important for cell migration [34, 35] and has been shown to lead to metastasis in breast and colon cancer [36, 37]. Focal adhesion disassembly in the cells was examined by treating them with nocodazole which stimulates focal adhesion formation through interfering with microtubule polymerization and activation of RhoA GTPase. Washout of nocodazole initiates the microtubule polymerization and re-activation of focal adhesion disassembly [38]. As shown in Figure 4C, treatment of control cells with nocodazole induced focal adhesion formation; focal adhesion disassembly was observed as early as 30 minutes after washing the cells. In contrast, *GATA2* gene silenced cells showed persistent focal adhesion complexes even after 120 minutes of nocodazole washout. This indicates that the *GATA2* gene plays an important role in promoting focal adhesion disassembly.

### Changes in the transcriptome induced by GATA2 gene silencing

**Figure 5:**
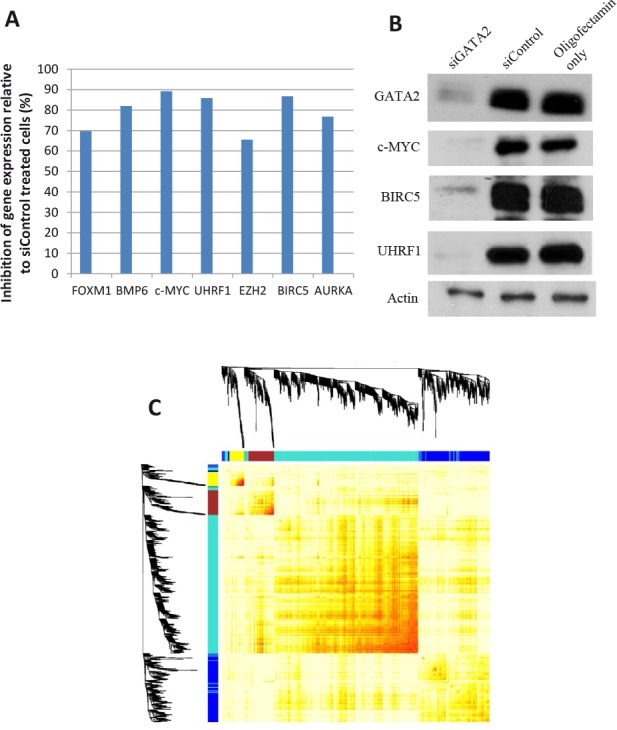
Microarray gene expression data A number of genes were validated for gene expression changes by A, qRT-PCR and/or B, Western blot analysis. C, weighted gene co-expression network analysis to identify genes that are potentially regulated by *GATA2*. The 4 modules of genes showing high correlation, as indicated by unsupervised hierarchical clustering, were assigned different colors.

Control and *GATA2*-silenced cells were gene expression profiled using microarray technology (GSE49342). The *GATA2*-down-regulation led to changes in the gene expression pattern of LNCaP cells, i.e. to ~1650 down-regulated genes and ~850 up-regulated genes (>2 fold change in mRNA expression levels, FDR <0.05; Table 1). As depicted in Table 2, genes with a well-established role in cancer were down-regulated following *GATA2* gene silencing, including *FOXM1, c-MYC, UHRF1, EZH2, BMP6, AURKA*, and *BIRC5*. The down-regulation of some of these genes was validated using qRT-PCR or Western blot analysis (Fig. 5A, B).

**Table 1:** Number of genes showing expression changes following GATA2 gene knockdown

	FC >2; FDR< 0.5
Down-regulated genes	1652
Up-regulated genes	861

FC: fold change; FDR: false discovery rate. FDR was calculated by the Fischer's exact test and Benjamini-Hochberg (BH) multiple-test correction method

**Table 2:** Genes whose expression changed following GATA2 gene knockdown

	Gene	Fold change	Corrected p value
Metastasis	FOXM1	−2.84	0.004
	BMP6	−4.0	0.008
	CXCL12	−3.7	0.03
	F2R	−2.0	0.001
	EZH2	−2.24	0.004
	ITGA6	−2.5	0.002
	ITGB1	−3.4	0.01
	SNAI1	−3.1	0.01
Cell proliferation	MYC	−6.7	0.0003
	UHRF1	−8	0.0002
	BIRC5	−5.3	0.002
Cell cycle regulation	AURKA	−4.0	0.017
	AURKB	−4.5	0.02
Angiogenesis	VEGFB	−2.16	0.02
	ANG	−4.4	0.0004

Corrected p value was calculated by the Fischer's exact test and Benjamini-Hochberg (BH) multiple-test correction method.

### GATA2 functions as indicated by biostatistical analysis

To gain more detailed insights into the function of the *GATA2* gene in prostate cancer metastasis, we first identified a core set of 970 *GATA2*-relevant genes that were both significantly differentially expressed after *GATA2* gene silencing in LNCaP cells (Student's t-test; FDR < 0.05 and FC > 1.5) and whose expressions significantly correlated with those of the *GATA2* gene in the MSKCC Prostate Oncogenome Project (Pearson's correlation; correlation coefficient > 0.30, FDR < 0.01). Next, we subjected these 970 genes to weighted gene correlation network analysis (WGCNA) to identify clusters (modules) of highly correlated genes [39, 40]. Four modules of genes with high topological overlap were identified, where each module comprises a cluster of highly inter-connected genes (Fig. 5C). These modules are color-coded as turquoise, blue, brown, and yellow, and contain 569, 245, 95, and 53 genes, respectively. We investigated the clinical relevance of these modules in the MSKCC cohort by calculating the association of each module's eigengene value (a summary of gene expressions in that module) with prostate cancer status (primary or metastatic). Yellow and brown modules were found to be highly significantly, and the blue module moderately significantly, associated with prostate cancer metastasis. To investigate the biological relevance of these modules, gene function enrichment analysis was performed on the genes in each module annotated with their expression fold-change established in the *GATA2* silencing experiments. The data indicate that the brown module was significantly enriched for cell migration and tissue invasion functions and would be down-regulated after *GATA2* silencing, and that the yellow module was significantly enriched for the down-regulation of cell proliferation after *GATA2* silencing (Table 3; Supplementary Table 2).

**Table 3:** Module significance in prostate cancer metastasis and fonction annotation

Module	Total gene count	Significance of association with metastatic prostate cancer	Ingenuity Pathway Analysis (IPA)		
			Function annotation	Activation score	P-value
Brown	95	1.658e-14	Cell migration	−2.135 (↓)	2.74E-08
			Tissue invasion	−2.128 (↓)	141E-05
Yellow	53	1.07e-06	Cell proliferation	−3.788 (↓)	1.68E-04
Turquoise	569	1.80e-04	Activation of DNA endogenous promoter	−2.259 (↓)	2.51E-04
			Organism survival	−2.656 (↓)	2.85E-03
Blue	245	0.0856	Cell cycle progression	−2.141 (↓)	3.99E-03

P value was calculated using the Fischer's exact test and Benjamini-Hochberg (BH) multiple-test correction method.

## DISCUSSION

Metastatic prostate cancer is highly resistant to conventional therapy and is at present incurable [5, 6]. Development of therapeutic approaches specifically targeting prostate cancer metastasis-driving genes could lead to improved disease management. Such master regulatory genes may be identified by a comparison of gene expression profiles of non-metastatic and metastatic prostate cancer tissues. A major hurdle using this approach. however, is that primary prostate cancer samples, the usual source of non-metastatic prostate cancer cells, do not consist of pure non-metastatic cells, but also contain metastatic cells, making such a comparison not feasible. To overcome this problem, we previously developed a pair of metastatic LTL-313H and non-metastatic LTL-313B transplantable prostate cancer tissue xenograft lines in NOD-SCID mice from one patient's primary prostatic adenocarcinoma using subrenal capsule grafting technology [26]. This methodology tends to preserve important properties of the original cancers, including histopathology, chromosomal aberrations and gene expression profiles [41–43]. As well, the maintenance of the xenograft lines in the same type of graft site (under the kidney capsule) tends to ensure that their gene expression profiles are not subject to major micro-environmental differences. In view of the above, the transplantable LTL-313H and LTL-313B xenografts that were used in the present study appear to be highly clinically relevant and, as such, suitable for identification of prostate cancer metastasis-driving genes.

The present study was aimed at identifying genes whose elevated expression in prostate cancer is directly responsible for activation of an amplification cascade of downstream genes leading to the development of metastatic ability. To this end, we identified the highest differentially expressed genes in metastatic LTL-313H xenografts, compared to their non-metastatic LTL-313B counterparts, and used IPA's Upstream Regulator Analysis tool to predict upstream regulators accountable for the differential expression (Fig. 1). The finding that the predicted upstream regulatory genes (Supplementary Table 1) included *HIF1A, WT1*, and *EZH2* genes, reported to be associated with prostate cancer metastasis [44–46], suggests that the approach used had merit. We focussed on *GATA2* as a potential prostate cancer metastasis-driving gene, since this gene is well known as a master regulatory gene in the hematopoietic system with a role in tumorigenesis [22, 23]. That the *GATA2* gene may have an important role in prostate cancer metastasis is indicated by the effects of its silencing in prostate cancer LNCaP and C4-2 cell lines. In particular, the silencing of *GATA2* in LNCaP cells led to (i) a marked decrease in cell migration and tissue invasion (Fig. 3C–E), consistent with the biostatistical findings (Fig. 5C, Table 3), and (ii) disrupted focal adhesion disassembly (Fig. 4C), an important process in metastasis [34, 35]. The positive correlation found between elevated expression of the *GATA2* gene in clinical metastatic prostate cancers and poor patient prognosis (Fig. 2A–C), as also reported by others [47], indicates that the findings are clinically relevant, and that elevated expression of *GATA2* is associated with malignant progression of prostate cancer.

Further evidence for the *GATA2* gene being an important regulatory gene in prostate cancer is the finding that the silencing of the *GATA2* gene in LNCaP cells led to significantly changed expression of as many as 2400 genes (>2 fold change, FDR <0.05; Table 1). Induction of such a high number of gene expression changes by altered expression of only one transcription factor is rare, as indicated by a reported study of the effects of systematic repression of individual transcription factor genes on global gene expression [48]. The finding suggests that *GATA2* plays a critical role in the homeostasis of prostate cancer cell transcriptomes.

It is of interest that the genes whose expressions were altered by *GATA2* silencing included *FOXM1, BMP6, c-MYC, EZH2, BIRC5* and *UHRF1* (Table 2), i.e. genes reported to have a role in prostate cancer progression and metastasis, suggesting that they represent downstream genes activated by *GATA2* in the development of prostate cancer metastasis.

In studying downstream pathways of the *GATA2* gene in metastatic prostate cancer, identification via WGCNA of modules consisting of expression pattern-correlated genes (Table 3) will be particularly useful, since it pinpoints the GATA2-activated genes that are involved in the same biological processes or share regulatory mechanisms. Interestingly, the brown module identified not only consists of genes enriched in cell migration and tissue invasion, but also of genes whose functions are significantly correlated with metastatic prostate cancer. Follow-up experiments on the genes of the brown module (see Supplementary Table 2) appear to be warranted to get further insight into the role of the *GATA2* gene in prostate cancer metastasis.

Pioneer factors form a special class of transcription factors that can associate with compacted chromatin to facilitate the binding of additional transcription factors. As such, they could play an important role in the formation of gene network cascades. Recently, GATA2 was identified as a pioneer factor in the regulation of AR target gene expression [30–32]. The present study, however, did not show evidence that AR-mediated signalling in LNCaP cells was among the top pathways affected by *GATA2*-silencing, indicating that the AR pathway does not constitute a major pathway of *GATA2* in prostate cancer metastasis. Further studies in this area appear to be warranted.

In conclusion, the findings of the present study suggest that the *GATA2* gene could represent a prostate cancer metastasis-driving gene, but further experimental proof is needed. If confirmed, the *GATA2* gene would represent a new and important target for therapy of metastatic prostate cancer.

## MATERIALS AND METHODS

### Materials

Chemicals, solvents and solutions were obtained from Sigma-Aldrich, Oakville, ON, Canada, unless otherwise indicated.

### Cell culture

Human LNCaP and C4-2 prostate cancer cells were obtained from the American Type Culture Collection (ATCC). Monolayer cultures were maintained in RPMI-1640 (Gibco BRL, Gaithersburg, MD) supplemented with 10% fetal bovine serum (FBS) as previously reported [49].

### Identification of upstream regulatory genes

The gene expression microarray dataset of xenograft lines LTL-313H vs LTL-313B [27] was normalized with Z-score transformation [50]. Genes showing changes in expression (with a z-ratio>0.5) were analyzed for identification of upstream regulatory genes using the Ingenuity Upstream Regulator Analysis tool (IPA; Ingenuity Systems Inc., Redwood City, CA). The gene expression data are accessible through GEO: GSE41193 (http://www.ncbi.nlm.nih.gov/geo/query/acc.cgi?acc=GSE41193).

### Clinical relevance analysis

Gene expression profiles and clinical information of MSKCC prostate adenocarcinomas [28] were downloaded from the CBio Cancer Genomics Portal website [29], and correlations were sought between poor prognostic factors of the patients and the relative expression levels of *GATA2* in their prostate cancer tissues. Statistical significance was established using the Student's t-test.

### Tissue microarray (TMA) construction and immunohistochemistry

A total of 359 specimens [60 benign prostate tumors, 137 primary tumors with no lymph node metastasis, 30 primary tumors with lymph node metastasis, 65 neo-adjuvant-treated primary tumors, 67 castration-resistant prostate cancers (CRPC)] were obtained from the Vancouver Prostate Centre Tissue Bank with written informed patients' consent and institutional study approval. All samples had been obtained through radical prostatectomy except CRPC samples that had been obtained through transurethral resection of prostate (TURP). The TMA construction has previously been described [51]. Immunohistochemical staining with polyclonal rabbit antibody against GATA2 (Cat No NBP1-82581, Novus Biological, Littleton, CO) was conducted using a Ventana autostainer (model Discover XT; Ventana Medical System, Tucson, AZ) with an enzyme-labelled biotin-streptavidin system and a solvent-resistant DAB Map kit (Ventana). Values on a four-point scale were manually assigned to each immunostaining by a pathologist. Descriptively, 0 represents no staining by any tumor cells, 1 represents a faint or focal, questionably present stain, 2 represents a stain of convincing intensity in a minority of cells and 3 a stain of convincing intensity in a majority of cells.

### siRNA transfection

Small interfering RNA (siRNA) targeting *GATA2* (siGATA2) and negative control (scrambled) siRNAs were purchased from Dharmacon (Cat No's J009024-07-0005 and D001810-10-05, Chicago, IL). To silence *GATA2* expression *in vitro*, cells were transfected with siGATA2 (30 nM; 48 or 72 hours) in oligofectamin reagent (Invitrogen, Carlsbad, CA) following the manufacturer's instructions.

### Western blotting

Cells were lysed using RIPA lysis buffer (50 mM Tris-Cl pH 7.4, 150 mM NaCl, 1% Igepal, 0.5% Na-deoxycholate, 0.1% SDS) supplemented with a protease inhibitor cocktail (Roche, Nutley, NJ). Whole-cell lysates (20 μg), whose protein concentrations were determined using the BCA protein assay (Thermo Fisher Scientific, Fremont, CA), were run on 8% SDS Polyacrylamide gel for Western blotting. The following antibodies were used: anti-GATA2 (Novus Biological), anti-c-Myc (Cat No Sc-40, Santa Cruz Biotechnology, Santa Cruz, CA), anti-UHRF1 (Cat No MABE308, Millipore, Billerica, MA), anti-BIRC5 (Cat No 2808, Cell Signaling Technology, Danvers, MA) and anti-β-actin (Cat No L002, Epitope Biotech Inc., Burnaby, BC, Canada).

### MTS cell proliferation assay

Cells were seeded onto 96-well culture plates (3000/well) and MTS (Promega, Madison, MI) was used to determine the cell populations following the manufacturer's instructions. The absorbance of formazan (reduced MTS) at 490 nm was measured daily. Statistical significance was established using the Student's t-test.

### Wound healing cell migration assay

Cells (8xl0^5^) that had been transfected with siGATA2 or siControl in maintenance medium were seeded onto 24-well culture plates and incubated at 37C in a 5% C0_2_ incubator; following cell attachment, the medium was changed to serum-free medium. The next day, a wound was created in the middle of a confluent cell monolayer using a pipette tip. Cell debris was removed by washing with lxPBS (2–3 times) and the cells further incubated in RPMI medium supplemented with FBS (10%). Photographic images were taken using a Zeiss Axiovert 200M microscope (Carl Zeiss Inc., Oberkochen, Germany) immediately after generating the wound, and after 8 and 24 hours of further incubation [52]. The cell-recovered areas at 8 hours were measured to estimate the extent of cell migration using Adobe Photoshop (Adobe, San Jose, CA). Statistical significance was established using the Student's t-test.

### Modified Boyden Chamber assays

Migration and matrigel invasion of cells, treated with siGATA2 or siControl, were determined using modified Boyden Chambers (BD Bioscience) following the manufacturer's instructions. After a 20-hr incubation at 37°C in a 5% C0_2_ incubator, both upper and lower chambers were washed twice with lxPBS. Dissociation buffer (300 μL; Trevigen, Gaithersburg, MD) containing calcein AMS (12.5 mM; Trevigen) was added to lower chambers for a further 1-hr incubation. Fluorescence (485 nm excitation, 520 nm emission) of cell suspensions (100 uL) was determined using 96-well plates and an Infinite F500 fluorometer (Tecan, Männedorf, Switzerland). The number of cells migrated/invaded to the bottom chambers was derived from the fluorescence reading using a standard curve. Statistical significance was established using the Student's t-test.

### Focal adhesion disassembly assay and immunofluorescence staining

Serum-starved (overnight) cells on cover slips were incubated with nocodazole (10 μM; Sigma) for 4 hours [38]. The cells were then washed with serum-free medium (3x) to remove the drug and the cover slips collected at various time intervals. Cells were fixed with 4% paraformaldehyde in PBS for 10 min and then permeabilized with 0.5% Triton-X100 in PBS for 10 min. For immunofluorescence staining, cells were stained with anti-vinculin (Cat No V4505, Sigma), and anti-GATA2 (Novus Biological); secondary antibodies were obtained from Jackson Immuno Research (West Grove, PA). Slides were mounted using DAPI mounting solution (Vector Laboratories, Burlingame, CA) and viewed using a LSM 780 Confocal Microscope (Carl Zeiss Inc.).

### Total RNA isolation and quantitative Real-Time PCR (qRT-PCR)

Total RNA was isolated from cultured cells using the RNeasy mini kit (Qiagen Inc., Hilden, Germany) following the manufacturer's instructions. Total RNA (1 μg) was used to synthesize cDNAs using a QuantiTect Reverse Transcription Kit (Qiagen Inc.). qRT-PCR reactions using KAPA SYBR Fast Universal (Kapa Biosystems, Woburn, MA) were performed in a ViiA 7 Real-Time PCR system (Applied Biosystems, Foster City, CA). The primer sequences used can be found in the Supplementary Table 3.

### Gene expression data profiling

The quality of the RNA samples was checked with the Agilent 2100 Bioanalyzer and NanoDrop ND-2000 UV-VIS spectrophotometer. Only samples with RNA Integrity Number (RIN) ≥8.0, A260/280 OD values between 1.8 and 2.0 and an A260/A230 OD value of 2.0 were used for one-color labelling using Agilent's One-Colour Microarray-Based Gene Expression Analysis Low Input Quick Amp Labelling v6.0 (Agilent Technologies, Santa Clara, CA). Total RNA (100 ng) was used to generate cyanine-3-labelled cRNA. Four replicates from each sample group (siGATA2- or siControl-treated cells) were hybridized on Agilent SurePrint G3 Human GE 8x60K Microarray v2 (Design ID 039494). Arrays were scanned with an Agilent DNA Microarray Scanner at a 3 μm scan resolution and data were processed with Agilent Feature Extraction 11.0.1.1. Processed signals were quantile normalized with Agilent GeneSpring 12.0. The data have been deposited in NCBI's Gene Expression Omnibus [53] and are accessible through GEO: GSE49342 (http://www.ncbi.nlm.nih.gov/geo/query/acc.cgi?acc=GSE49342).

### Gene expression data analysis

Microarray gene expression data were filtered for improved quality prior to downstream analysis. Specifically, probes without corresponding gene annotations and probes without detectable expression levels (less than 3 in log2 scale) were removed. Significantly differentially expressed genes after siGATA2 treatment were selected based on the Student's t-test with multiple test correction (FDR < 0.05) and a fold difference in mean probe expression ≥ 2.0 in the siGATA2-treated samples relative to the control samples.

### Weighted gene co-expression network analysis (WGCNA)

In the WGCNA [39, 40] used, a gene network was first constructed by treating each gene as a node and assigning a weighted edge between each pair of nodes based on the strength of their co-expression across the MSKCC cohort of 132 primary and 18 metastatic samples as calculated by Pearson's correlation. Correlations found were used to calculate topological overlap measure (TOM). Pairs of genes with high topological overlap were filtered. Highly inter-connected gene clusters, known as modules, were identified using unsupervised hierarchical clustering on the 1-TOM distance values with a dynamic tree-cutting process. The significance of resulting highly inter-connected gene modules was investigated in two ways. First, the module eigengene value was checked for association with clinical outcome. Second, the component genes of each module were used in gene enrichment analysis using IPA software to determine biological relevance. Statistical over-representation of functions was calculated using the Fischer's exact test and Benjamini-Hochberg (BH) multiple-test correction method, where functions with a BH-adjusted p-value <0.05 were considered significant.

## Supplementary Figure and Tables




