# Supplementary material for: GATA2 as a potential metastasis-driving gene in prostate cancer
Source: Oncotarget. 2014 Jan 12;5(2):451–61. doi: 10.18632/oncotarget.1296 (PMC3964220; doi:10.18632/oncotarget.1296)
Supplement: Supplementary file 1 [file oncotarget-05-451-s001.pdf]

Supplementary Fig. 1A

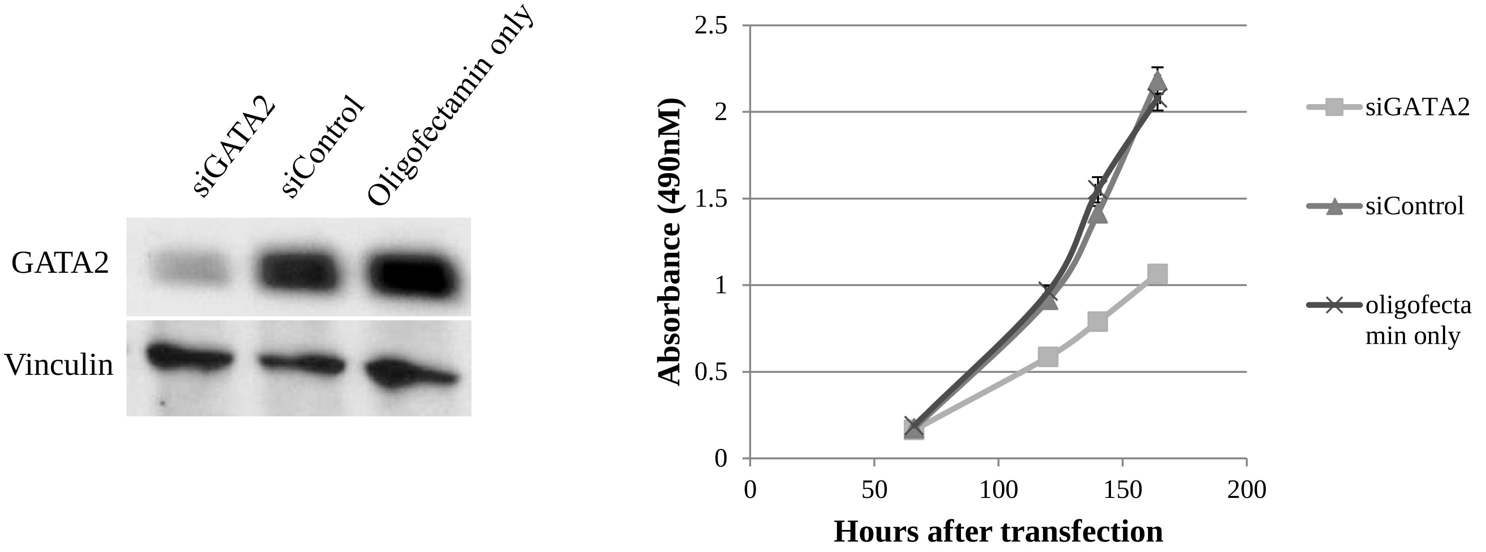

Supplementary Fig. 1B

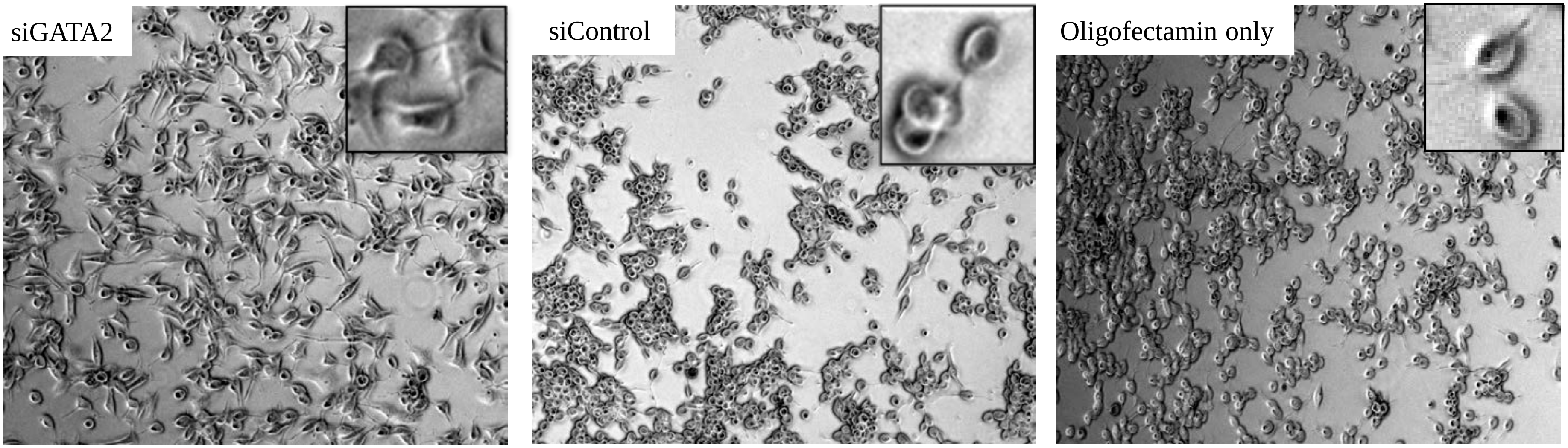

Supplementary Figure 1: A) *GATA2* silencing markedly reduced proliferation of C4-2 prostate cancer cells. B) *GATA2* silencing induced morphological changes in C4-2 prostate cancer cells. The normally rounded C4-2 cells became flat and spindle-like.
